# Prevalence and correlates of PrEP use and missed PrEP doses due to alcohol among sexually active adults and sexual minority men in San Francisco

**DOI:** 10.1016/j.drugalcdep.2025.112960

**Published:** 2025-11-17

**Authors:** Michael Deynu, Alexandrea Dunham, Jerry John Ouner, Glenn-Milo Santos

**Affiliations:** a University of California San Francisco, Department of Family Health Care Nursing, San Francisco, CA 94102, United States; b San Francisco Department of Public Health, San Francisco, CA 94102, United States; c University of California San Francisco, Department of Community Health Systems, San Francisco, CA 94102, United States

**Keywords:** Pre-exposure prophylaxis, Sexual minority men, Alcohol, Cocaine, Sexually Transmitted infections, HIV

## Abstract

**Background:**

Alcohol use is linked with HIV-associated sexual behaviors and new HIV infections, and individuals who drink alcohol may benefit from Pre-Exposure Prophylaxis (PrEP) to prevent HIV. We evaluated the prevalence and correlates of current PrEP use and missed PrEP doses due to alcohol.

**Methods:**

We included 604 HIV-negative participants (i.e., those eligible for PrEP) in San Francisco. Primary outcomes were current PrEP use and missed PrEP doses due to alcohol use in the past 3 months. Modified Poisson regression models with robust standard errors were fitted separately for each exposure while controlling for age, gender, and race/ethnicity.

**Results:**

We enrolled a diverse sample comprising 56 % cisgender male, 42 % cisgender female, and 2 % transgender participants, and 33 % who were sexual minority men (SMM). Mean age was 39.45 years (SD=12.50). Current PrEP use was 21 % and missed PrEP doses due to alcohol were reported by 51 % of the sample who used PrEP. Factors associated with greater odds of PrEP use overall include: having anal intercourse while under the influence of alcohol (14.57 [7.17,29.60], p < 0.001) and any STIs (1.94 [1.40,2.69], p < 0.001), all in the past 3 months. Each additional day of cocaine use was associated with lower odds of PrEP use (0.93 [0.89,0.98], p = 0.01). Condomless anal intercourse under the influence of alcohol were associated with greater odds of missing PrEP doses (17.47 [7.05–43.30], p < 0.001). Among SMM, cocaine use was associated with lower odds of PrEP use (0.91 [0.86–0.97], p = 0.03, while having condomless vaginal intercourse while under the influence of alcohol was associated with missing PrEP doses due to alcohol (2.07 [1.11,3.82], p = 0.02) in the last 3 months.

**Conclusions:**

PrEP prevalence is low, with more than half the participants reporting missed PrEP doses due to alcohol use, which was associated with engaging in sexual activity while under the influence of alcohol. Furthermore, PrEP use is associated with increased alcohol use and recent STIs diagnoses. These findings can help optimize interventions to increase PrEP use and adherence among sexually active adults who drink alcohol, including SMM. Further, the findings also highlight the need for integrating alcohol use interventions into PrEP care.

## 1. Introduction

Pre-exposure prophylaxis (PrEP) is one key strategy in HIV prevention (Chou et al., 2019; McCormack et al., 2016). Although efficacious, PrEP uptake among populations who may benefit from it has generally been low (Kamitani et al., 2020, 2018; Parsons et al., 2017; Sullivan et al., 2020), including individuals engaged in unhealthy alcohol use (Fletcher et al., 2025). For instance, sexually active individuals who drink alcohol may have high risk for HIV, as heavy drinking patterns and concurrent alcohol use during sexual encounters have been independently associated with HIV transmission (Hutton et al., 2019; Oldfield and Edelman, 2021). Examining factors that are associated with PrEP use among sexually active adults who drink alcohol can help develop approaches to increase PrEP uptake among this population at risk for HIV.

Moreover, alcohol has been identified as a potential barrier to PrEP use and adherence (Krakower et al., 2019; Shuper et al., 2020, 2022). A scoping review with 53 studies on PrEP use among individuals with unhealthy alcohol use reported disruptions related to alcohol use along the PrEP care continuum, from PrEP awareness to adherence (Oldfield and Edelman, 2021). Similarly, among individuals who use alcohol, younger age, club drug use, missing PrEP appointments, engaging in condomless sexual activity, forgetfulness and depressive symptoms were associated with PrEP non-adherence (Shuper et al., 2024). Also, low HIV risk is associated with non-use of PrEP among individuals who use substance (Verinumbe et al., 2024). However, limited studies have examined factors that are associated with lapses in PrEP adherence due to alcohol use while also engaging in sexual activity. Understanding these factors can help identify individuals who could benefit from PrEP adherence support strategies or other PrEP regimens, including long-acting injectables or event-driven PrEP use. To address these gaps in the literature, we evaluated the prevalence and correlates of current PrEP use and missed PrEP doses due to alcohol outcomes among sexually active adults who drink alcohol.

## 2. Methods

### 2.1. Study setting, design, and participant recruitment

We conducted a secondary analysis of a screening data among participants assessed for eligibility for a randomized clinical trial conducted to test the efficacy of kudzu, an herbal supplement, in reducing alcohol use among individuals with AUD in San Francisco (clinicaltrials.gov # NCT03709043). The data for the parent study were collected between September 2018 and July 2023. To ensure eligibility for the parent study, study participants were required to have 1. AUD. 2. 18 years or older, 3. Bay Area residents, 4. consumed alcohol, 5. reported any anal or vaginal intercourse, or diagnosed with sexually transmitted infection (STI) in the past 3 months, 6. were HIV-negative by self-report, and 7. provided consent to be screened for the trial. For the screening data used for this study, prospective participants were screened over the phone by research staff who confirmed that participant’s name and contact information were not duplicate responses in the study recruitment database.

Further, during the interview, demographic questions on age, gender, sex at birth, preferred pronouns, and whether participants were living in the Bay Area were used to determine and validate eligibility. Therefore, the data analyzed in this study were restricted to participants who were 1. 18 years or older, 2. Bay Area residents, 3. consumed alcohol, 4. reported any anal or vaginal intercourse, or diagnosed with sexually transmitted infection (STI) in the past 3 months, 6. were HIV-negative by self-report, and 6. provided consent to be screened for the trial. For this study, we included a sample of 604 sexually active adults, 198 of whom were SMM. The sub-sample of SMM was defined as cisgender men who reported having any anal intercourse with men, regardless of vaginal intercourse in the last 3 months (because sexual behaviors could be fluid and individuals self-identifying as sexual minority men may engage in vaginal intercourse for a variety of reasons).

### 2.2. Ethical approval

The University of California San Francisco, San Francisco (UCSF) institutional review board (IRB) approved this study (IRB approval number # 18–25073).

### 2.3. Measures

#### 2.3.1. Outcomes

PrEP use was assessed using the question, “Are you currently taking pre-exposure prophylaxis (PrEP)?” (yes/no). Missed PrEP doses due to alcohol were assessed among individuals who use PrEP using the following question: “In the past 3 months, did you miss any PrEP doses due to drinking alcohol?” (yes, no).

#### 2.3.2. Correlates

We included measures on the number of drinks containing alcohol per day, number of drinking days, number of times under the influence of alcohol while having vaginal intercourse, number of times under the influence of alcohol while having anal intercourse, cocaine use, and sexually transmitted infection (STIs) diagnoses in the last 3 months. Heavy drinking was defined as consuming 5 or more drinks containing alcohol on a single occasion for men and consuming 4 or more drinks on a single occasion for women. For the purposes of this analysis, heavy drinking was defined as consuming 5 or more drinks and 4 or more drinks containing alcohol on a single occasion for men and women respectively.

### 2.4. Data analysis

We fitted separate Poisson regression models with robust standard errors (Gnardellis et al., 2022; Sedgwick, 2014; Zou, 2004) for each outcome and exposure, while we controlled for age, gender, and race/ethnicity. The separate modelling approach ensured we avoided reporting effect estimates for the exposure (independent variables) and covariates of interest from the same model, which may be misleading and result in incorrect interpretations (Bandoli et al., 2018; Westreich and Greenland, 2013). The Poisson regression model was selected because odd ratios may be unintuitive and potentially misinterpreted as risk ratios or they may overestimate the effect size when the outcome prevalence is greater than 10 % or common (Talbot et al., 2023; Zou, 2004). Age, participant gender, and race/ethnicity were included as covariates that are potential confounders based on the literature (Groenwold et al., 2011).

Additionally, we conducted a separate analysis on a sub-sample of SMM (i.e., cisgender men who have sex with men). This is because we hypothesized that there may be potential differences in PrEP uptake among SMM compared to the larger sample. The SMM models adjusted for age and race/ethnicity. Potential correlates were selected into the Poisson regression with robust standard errors model based on statistical significance (p < 0.05), theoretical and clinical knowledge and public health relevance. Further, analysis were also conducted among cisgender males and females and cisgender SMM and transgender individuals to estimate the correlates of PrEP use and Missed PrEP doses due to alcohol among these populations. All analyses were conducted using STATA version 18.0 (StataCorp, 2023).

## 3. Results

### 3.1. Demographic characteristics

Participant characteristics are summarized in Table 1. The mean age was 39.45 years (SD=12.50), and the mean number of days cocaine was used was 1.94 (SD=5.65). Among the overall sample, 56.4 % of the participants identified as cisgender male, 41.5 % identified as cisgender female, while 2.0 % identified as a transgender individual. More than half (58.61 %) of the sample identified as racial and ethnic minorities. Among the SMM sub-sample, the mean participant age was 38.79 (SD=12.21) years. Also, nearly 58 % of SMM identified as racial and ethnic minority individuals.

The majority, 63.25 % of the overall sample reported drinking four or more drinks containing alcohol per day while 68.18 % of SMM reported drinking four or more drinks containing alcohol per day, all in the last 3 months. Further, 88.89 % of SMM reported having anal intercourse with condom while under the influence of alcohol.

### 3.2. Distributions of PrEP use and missed PrEP in the overall sample

PrEP prevalence was 21.0 %, while 51.0 % of participants on PrEP reported missed PrEP doses due to their alcohol use. PrEP use differed between demographic and behavioral characteristics. For example, 73.4 % of cisgender males compared to 8.6 % of transgender males were using PrEP (p < 0.001). Furthermore, 83.0 % of the sample who reported drinking an average of four or more drinks on drinking days missed PrEP doses (p = 0.02) compared to 2 % of those who consumed less than one drink on drinking days. Further, each additional day of cocaine use was associated with PrEP use (p = 0.01). Also, 60.0 % and 54.0 % of SMM who reported having vaginal intercourse and condomless vaginal intercourse while under the influence of alcohol, respectively, missed PrEP (p < 0.001) compared to individuals who do not drink alcohol (Table 1).

### 3.3. Correlates of PrEP use and missed PrEP doses due to alcohol in the overall sample

The results of the adjusted Poisson regression with robust standard errors on correlates of current PrEP use and missed PrEP doses due to alcohol in the overall sample are shown in Table 2. We found lower current PrEP use among individuals reporting vaginal intercourse (aRR=0.28 [95 % CI:0.21,0.39], p < 0.001) and condomless vaginal intercourse while under the influence of alcohol (aRR=0.61 [95 % CI:0.46,0.81], p < 0.001) in the last 3 months. In contrast, engaging in anal intercourse (aRR=14.57 [95 % CI:7.17,29.60], p < 0.001) and condomless anal intercourse (aRR=9.26 [95 % CI:5.42,15.81], p < 0.001) while under the influence of alcohol were associated with greater PrEP use, all in the last 3 months. Having anal intercourse while under the influence of alcohol (aRR=36.72 [95 % CI:9.32,144.70], p < 0.001) and condomless anal intercourse while under the influence of alcohol (aRR=17.47 [95 % CI:7.05,43.30], p < 0.001), were associated with greater missed PrEP doses due to alcohol, all in the last 3 months. Being diagnosed with gonorrhea (aRR=1.75 [95 % CI:1.18,2.61], p=0.01) or chlamydia (aRR=1.95 [95% CI:1.34,2.84], p < 0.001), as well as any STIs (aRR=1.94 [95 % CI:1.40,2.69], p < 0.001), were associated with higher PrEP use.

### 3.4. Correlates of PrEP use and Missed PrEP doses due to alcohol among SMM

The results of the adjusted Poisson regression with robust standard errors on correlates of current PrEP use and missed PrEP doses due to alcohol among SMM are shown in Table 2. Each additional day of cocaine use (aRR=0.91 [95 % CI:0.86,0.97], p = 0.00 and having vaginal intercourse while under the influence of alcohol (aRR=0.57 [95 % CI:0.40,0.82], p < 0.001 were associated with lower PrEP use, all in the last 3 months. Conversely, being diagnosed with gonorrhea (aRR=1.66 [95% CI:1.15,2.39], p= 0.01), chlamydia (aRR=1.80 [95% CI:1.31,2.46], p < 0.001), as well as any STIs (aRR=1.66 [95 % CI:1.21,2.26], p = 0.02 were associated with greater PrEP use. Further, having condomless vaginal intercourse while under the influence of alcohol was associated with higher missed PrEP doses due to alcohol among SMM in the last 3 months, (see Table 2).

### 3.5. Correlates of PrEP use and Missed PrEP doses due to alcohol among Cisgender males and females

Table 3 summarizes results of the adjusted Poisson regression with robust standard errors on the correlates of PrEP use and Missed PrEP doses due to alcohol among Cisgender men and women. Increasing cocaine use (aRR=0.92 [95 % CI:0.87,0.98], p = 0.01), having vaginal intercourse (aRR=0.25 [95 % CI:0.18,0.36], p < 0.001, and having condomless vaginal intercourse (aRR=0.57 [95 % CI:0.43,0.77], p < 0.001) while under the influence of alcohol were associated with lower PrEP use among Cisgender men and women.

Having condomless anal intercourse while under the influence of alcohol was associated with greater PrEP use (aRR=11.46 [95 % CI:6.29,20.86], p < 0.001) in the last 3 months. Also, having condomless anal intercourse while under the influence of alcohol was associated with greater missed PrEP doses due to alcohol (aRR=21.55 [95 % CI:7.91,58.68], p < 0.001), in the last 3 months.

### 3.6. Correlates of PrEP use and Missed PrEP doses due to alcohol among Cisgender SMM and transgender individuals

The results of the adjusted Poisson regression with robust standard errors on the correlates of PrEP use and Missed PrEP doses due to alcohol among Cisgender SMM and Transgender participants are presented in Table 3 below. Increasing cocaine use (aRR=0.92 [95 % CI; 0.88,0.97], p = 0.00) and having vaginal intercourse while under the influence of alcohol (aRR=0.59 [95 % CI:0.43,0.82], p = 0.00) were associated with lower PrEP use respectively, all in the last 3 months. However, having condomless vaginal intercourse (aRR=2.02 [95 % CI:1.14,3.59], p = 0.02 while under the influence of alcohol was associated with greater missed PrEP doses due to alcohol in the last 3 months.

## 4. Discussion

Among our sample of sexually active adults who drink alcohol, while PrEP prevalence was low overall (21.0 %), it was higher among SMM in the sample (45.5 %). This finding is broadly consistent with other studies that have previously noted that PrEP remains underutilized, especially among groups outside of SMM (Friedman et al., 2019; Kamitani et al., 2020; Sevelius et al., 2020; Siegler et al., 2018). We also found that 51.0 % of participants in the overall sample and 38.9 % of SMM on PrEP reported missed PrEP doses due to alcohol. This is consistent with other studies, which previously observed disruptions in PrEP adherence associated with alcohol use across study populations, including SMM (Kalichman et al., 2014; Oldfield and Edelman, 2021; Tran et al., 2014).Our finding showed sub-optimal PrEP use among sexually active individuals and SMM and underscores the need for urgent public health interventions aimed at increasing PrEP use among sexually active individuals including those with concurrent alcohol use. Public health interventions that focuses on expanding access to PrEP care and the implementation of innovative digital service delivery models are warranted to increase PrEP use among these population.

Additionally, we found greater odds of current PrEP use among participants who engaged in anal intercourse and condomless anal intercourse while under the influence of alcohol and who had been diagnosed with STI in the overall sample in the last 3 months. These findings are encouraging because they may suggest a higher uptake of PrEP among those engaging in behaviors that have been linked to HIV/STI risks. Our finding is consistent with prior studies, which found that SMM engaged in sexual behaviors are more likely to use PrEP (Goedel et al., 2018; Hardy et al., 2022). Notably, we observed lower odds of PrEP use among those who reported engaging in vaginal intercourse while under the influence of alcohol in both the overall sample and among SMM. Our finding is consistent with prior studies that reported low PrEP uptake among cisgender women and those engaged in vaginal intercourse (Blumenthal et al., 2021).

These findings point to a potential gap in PrEP access and use among individuals who drink alcohol while engaging in vaginal intercourse and underscores the need for interventions to increase PrEP use among these individuals. Further, innovative targeted PrEP care counseling, peer support systems, harm reduction counseling (focusing on alcohol and substance use) (Teixeira da Silva et al., 2021), motivational interviewing and behavioral support strategies (Chan et al., 2021; Dale, 2020) may offer potential benefits for these population as well as their sexual partners in increasing PrEP use. Additionally, healthcare workers could encourage sexual partner participation at counseling sessions to enhance attainment of mutual goals related to PrEP use to prevent HIV (Malotte, 2013) among these populations particularly for those engaged in vaginal intercourse while under the influence of alcohol. Further, integrated sexual health and STI screening services, strategies to addressing social barriers and provider trainings on the need for non-judgmental screening and service delivery models may offer potential benefits to reducing individual barriers and enhancing PrEP adherence among these individuals and their sexual partners.

Moreover, we observed greater odds of missed PrEP doses due to alcohol use among participants who reported engaging in anal intercourse while drinking and engaging in condomless vaginal intercourse while drinking, both in the overall sample and among SMM in the last 3 months. These findings broadly align with prior studies that have observed that alcohol use can function as a barrier to PrEP adherence (Oldfield and Edelman, 2021). A myriad of reasons are posited to contribute to missing PrEP doses due to alcohol use. For example, beliefs associated with potential toxicity interaction between alcohol and antiretroviral medications (Kalichman and Eaton, 2017) and forgetfulness, particularly when intoxicated (Beesham et al., 2022; Qu et al., 2018; Strong et al., 2024), have been previously observed.

We also found that each additional day of cocaine use was associated with lower odds of current PrEP use among SMM. This finding is corroborated in other studies that reported cocaine use was associated with lower PrEP adherence, including objective measures using lower serum tenofovir concentrations (Hojilla et al., 2019). Marcus and colleagues reported similar findings where patients with a history of drug use were almost twice as likely to disengage from PrEP (Marcus et al., 2016). Another study found that individuals engaged in polysubstance use had lower PrEP use (Kalichman and Eaton, 2017; Tran et al., 2014; Velloza et al., 2020). It is also plausible that SMM who drink alcohol with more frequent cocaine use may have more significant delays in accessing clinical care and, therefore, face more disruptions in starting and persisting with PrEP (Hojilla et al., 2019). These findings suggest that people who use stimulants like cocaine and alcohol may benefit from expanded efforts to link and retain them in the PrEP care continuum.

We observed similar findings when the analyses were stratified by cisgender males and females, and by cisgender SMM and Transgender individuals. Altogether, the results highlight the influence of behavioral factors on PrEP use and missed PrEP doses due to alcohol among our sample and underscores the importance of implementing concerted public health efforts aimed at increasing PrEP use and reducing missed PrEP doses due to alcohol among this population.

## 5. Study limitations

Our study has several limitations. Firstly, our outcomes were based on self-report, which could have led to social desirability and recall biases (Agot et al., 2015). Further, the cross-sectional nature limits our ability to establish causation. Since our sample was restricted to the San Francisco Bay Area, we are limited in our ability to generalize our findings. Also, we dichotomized our race/ethnicity variable due to low sample, which could have led to the loss of information related to participants’ unique experiences.

Finally, because our study relied on baseline data from an intervention study, the measures for correlates of PrEP use and missed PrEP doses available were limited. For instance, due to unavailability of data on baseline sexual behaviors, we were unable to examine their confounding effect in the observed associations in our model. Future studies could include these to examine their effect. Similarly, additional studies with more extensive assessments/measures can help further elucidate factors that affect PrEP use and adherence among sexually active adults who drink alcohol.

## 6. Conclusions

This study underscores the influence of concurrent sexual behaviors and alcohol use, as well as STI and cocaine use, on PrEP utilization and adherence. Our findings, together with prior studies, highlight the need for strategies that focus on the integration of alcohol use interventions into PrEP care while also expanding PrEP access among people who drink alcohol– especially those engaging in vaginal sex and using stimulants. We also identified subgroups of sexually active adults who drink alcohol and may benefit from interventions to increase PrEP adherence and/or reduce missed PrEP due to alcohol. Additionally, offering on-demand PrEP dosing or long-acting injectable PrEP may address these subgroups’ low uptake and missed doses of oral PrEP. Ultimately, these results support the need to develop and expand interventions to increase PrEP uptake and PrEP adherence among key populations, including sexually active adults and SMM who drink alcohol.

## Figures and Tables

**Table 1:** Participant characteristics and bivariate distribution of PrEP use and missed PrEP due to alcohol in the overall sample (N = 604), and among Sexual Minority Men, SMM (N = 198).

Characteristics	Overall Sample							Sexual Minority Men						
	Frequency n(%)	Current PrEP Use No(n = 476)	Yes (n = 128)	Sig.	Missed PrEP Doses No(n = 63)	Yes(n = 65)	Sig.	Frequency n(%)	Current PrEP No(n = 108)	Yes(n = 90)	Sig.	Missed PrEP No(n = 55)	Yes(n = 35)	Sig.
**Age** (years) Mean (SD)	39.45(12.50)	40.29(12.74)	36.30(11.02)	0.001	35.97(10.10)	36.62(12.29)	0.74	38.79(11.10)	40.06(12.21)	37.27(11.62)	0.10	36.16(10.51)	39.0(13.15)	0.26
**Participant Gender**				<0.001			<0.001							
Cisgender Male	341(56.46)	247(51.9)	94(73.4)		59(94.0)	35(54.0)								
Cisgender Female	251(41.56)	228(47.9)	23(18.0)		0(0.0)	23(35.0)								
Transgender	12(1.99)	1(0.2)	11(8.6)		4(6.0)	7(11.0)								
**Racial and Ethnic Minority**				0.31			<0.001				0.16			0.02
No	250(41.39)	202(42.4)	48(37.5)		36(57.0)	12(18.0)		84(42.42)	41(38.0)	43(47.80)		32(58.0)	11(31.0)	
Yes	354(58.61)	274(57.6)	80(62.5)		27(43.0)	53(82.0)		114(57.58)	67(62.0)	47(52.20)		23(42.0)	11(69.0)	
**Number of drinks containing alcohol per day**				0.07			0.02				0.70			0.08
One per day or less	21(3.48)	18(3.8)	3(2.3)		2(3.0)	1(2.0)		6(3.03)	4(3.7)	2(2.2)		2(4.0)	0(0.0)	
Two to three drinks per day	201(33.28)	168(35.3)	33(25.8)		23(37.0)	10(15.0)		57(28.79)	29(26.9)	28(31.1)		21(38.0)	7(20.0)	
Four or more drinks per day	382(63.25)	290(60.9)	92(71.9)		38(60.0)	54(83.0)		135(68.18)	75(69.4)	60(66.7)		32(58.0)	28(80.0)	
**Number of days drink alcohol last 3 months**				0.00			0.83				0.14			0.82
Everyday	155(25.66)	132(27.7)	23(18.0)		10(16.0)	13(20.0)		41(20.71)	27(25.0)	14(15.6)		8(15.0)	6(17.0)	
Less than 7 days per week	408(67.55)	319(67.0)	89(69.5)		45(71.0)	44(68.0)		139(70.20)	74(68.5)	65(72.2)		41(75.0)	24(69.0)	
Less than 4 days per month	41(6.79)	25(5.3)	16(12.5)		8(13.0)	8(12.0)		18(9.09)	7(6.5)	11(12.2)		6(10.0)	5(14.0)	
**Vaginal intercourse while under the influence of alcohol in the last 3 months**				<0.001			<0.001				0.00			0.00
No	118(19.54)	53(11.1)	65(50.8)		50(79.0)	15(23.0)		111(56.06)	48(44.4)	63(70.0)		49(89.0)	14(40.0)	
Yes	486(80.46)	423(88.9)	63(49.2)		13(21.0)	50(77.0)		87(43.94)	60(55.6)	27(30.0)		6(11.0)	21(60.0)	
**Condomless vaginal intercourse while under the influence of alcohol in the last 3 months**				<0.001			<0.001				0.02			0.00
No	226(37.42)	158(33.2)	68(53.1)		51(81.0)	17(26.0)		128(64.65)	62(57.4)	66(73.3)		50(91.0)	16(46.0)	
Yes	378(62.58)	318(66.8)	60(46.9)		12(19.0)	48(74.0)		70(35.35)	46(42.6)	24(26.7)		5(9.0)	19(54.0)	
**Anal intercourse while under the influence of alcohol in the last 3 months**				<0.001			0.13							
No	326(53.97)	318(66.8)	8(6.2)		6(10.0)	2(3.0)								
Yes	278(46.03)	158(33.2)	120(93.8)		57(90.0)	63(97.0)		198(100.00)	108(100.00)	90(100.00)		55(100.00)	35(100.00)	
**Condomless anal intercourse while under the influence of alcohol in the last 3 months**				<0.001			0.15				0.07			0.77
No	361(59.77)	346(72.7)	15(11.7)		10(16.0)	5(8.0)		22(11.11)	16(14.8)	6(6.7)		4(7.0)	2(6.0)	
Yes	243(40.23)	130(27.3)	113(88.3)		53(84.0)	60(92.0)		176(88.89)	92(85.2)	84(93.3)		51(93.0)	33(94.0)	
**Cocaine use, number of days used in last 30 days (M, SD)**	1.94(5.65)	2.26(6.22)	0.76(2.20)	0.01	1.11(2.67)	0.42(1.57)	0.07	1.56(4.35)	2.31(5.64)	0.66(1.45)	0.01	0.85(1.56)	0.34(1.24)	0.10
**Diagnosed with Gonorrhea**				<0.001			0.00				0.03			0.01
No	584(96.69)	467(98.1)	117(91.4)		53(84.0)	64(98.0)		186(93.94)	105(97.2)	81(90.0)		46(84.0)	35(100.0)	
Yes	20(3.31)	9(1.9)	11(8.6)		10(16.0)	1(2.0)		12(6.06)	3(2.8)	9(10.0)		9(16.0)	0(0.0)	
**Diagnosed with Chlamydia**				<0.001			0.10				0.02			0.11
No	587(97.19)	470(98.7)	117(91.4)		55(87.0)	62(95.0)		188(94.95)	106(98.1)	82(91.1)		48(87.0)	34(97.0)	
Yes	17(2.81)	6(1.3)	11(8.6)		8(13.0)	3(5.0)		10(5.05)	2(1.9)	8(8.9)		7(13.0)	1(3.0)	
**Diagnosed with Syphilis**				0.14			0.29				0.82			0.16
No	594(98.34)	470(98.7)	124(96.9)		60(95.0)	64(98.0)		192(96.97)	105(97.2)	87(96.7)		52(95.0)	35(100.0)	
Yes	10(1.66)	6(1.3)	4(3.1)		3(5.0)	1(2.0)		6(3.03)	3(2.8)	3(3.3)		3(5.0)	0(0.0)	
**Any sexually transmitted infections(STIs)**				<0.001			<0.001				0.01			0.00
No	569(94.21)	461(96.8)	108(84.4)		46(73.0)	62(95.0)		176(88.89)	102(94.4)	74(82.2)		40(73.0)	34(97.0)	
Yes	35(5.79)	15(3.2)	20(15.6)		17(27.0)	3(5.0)		22(11.11)	6(5.6)	16(17.8)		15(27.0)	1(3.0)	
**Current PrEP Use**														
No	476(78.81)							108(54.55)						
Yes	128(21.19)							90(45.45)						
**Missed PrEP doses due to alcohol**														
No	63(49.22)							55(61.11)						
Yes	65(50.78)							35(38.89)						

NB: The variable for anal intercourse was used in defining the SMM sub-population. All participants reported anal intercourse.

**Table 2:** Adjusted Poisson regression with robust standard errors of the predictors of PrEP Use and missed PrEP due to Alcohol in the overall sample (N = 604) and Sexual Minority Men, SMM (N = 198).

Characteristics	Overall Sample						Sexual Minority Men (SMM)					
	Current PrEP Use			Missed PrEP due to Alcohol			Current PrEP Use			Missed PrEP		
	aRR	95% CI	Sig.	aRR	95 % CI	Sig.	aRR	95 % CI	P-value	aRR	95 % CI	Sig.
**Number of drinks containing alcohol per day in last 3 months**												
One (1) per day or less	ref	ref		ref	ref		ref	ref				
Two (2) – three (3) drinks per day	1.36	0.53–3.49	0.52	1.29	0.24–7.05	0.77	1.67	0.50–5.59	0.41			
4 or more drinks per day	1.75	0.70–4.34	0.23	3.17	0.62–16.25	0.17	1.61	0.48–5.38	0.44			
**Number of days drink alcohol last 3 months**												
Every day	ref	ref		ref	ref		ref	ref		ref	ref	
Less than 7 days/week	1.19	0.77–1.84	**0.43**	1.13	0.59–2.17	0.70	1.28	0.80–2.05	0.30	1.29	0.55-3.04	0.56
Less than 4 days per month	1.77	1.03–3.05	0.04	1.73	0.73–4.10	0.22	1.56	0.86–2.85	0.14	**2.26**	0.75-6.82	0.15
**Vaginal intercourse while under the influence of alcohol in the last 3 months**												
No	ref	ref		ref	ref		ref	ref		ref	ref	
Yes	0.28	0.21–0.39	**< 0.001**	0.71	0.41–1.24	0.23	0.57	0.40–0.82	**0.00**	1.80	0.97–3.34	0.06
**Condomless vaginal intercourse while under the influence of alcohol in the last 3 months**												
No	ref	ref		ref	ref		ref	ref		ref	ref	
Yes	0.61	0.46–0.81	<0.001	1.53	0.94–2.50	0.09	0.71	0.49–1.04	0.08	2.07	1.11–3.82	**0.02**
**Anal intercourse while under the influence of alcohol in the last 3 months**												
No	ref	ref		ref	ref							
Yes	14.57	7.17–29.60	**< 0.001**	36.72	9.32–144.70	**< 0.001**						
**Condomless anal intercourse while under the influence of alcohol in the last 3 months**												
No	ref	ref		ref	ref		ref	ref		ref	ref	
Yes	9.26	5.42–15.81	**< 0.001**	17.47	7.05–43.30	**<0.001**	1.84	0.94–3.60	**0.07**	1.94	0.50–7.63	0.34
**Cocaine use, number of days used in the last 30 days**	0.93	0.89–0.98	**0.01**	0.85	0.74–0.99	0.03	0.91	0.86–0.97	0.00	0.80	0.60–1.06	0.12
**Diagnosed with Gonorrhea**												
No	ref	ref		ref	ref		ref	ref				
Yes	1.75	1.18–2.61	**0.01**	0.29	0.06–1.45	0.13	1.66	1.15–2.39	**0.01**			
**Diagnosed with Syphilis**												
No	ref	ref		ref	ref		ref	ref				
Yes	1.26	0.63–2.52	0.51	0.67	0.21–2.18	0.51	1.07	0.48–2.38	0.88			
**Diagnosed with Chlamydia**												
No	ref	ref		ref	ref		ref	ref		ref	ref	
Yes	1.95	1.34–2.84	**0.001**	1.08	0.48–2.44	0.86	1.80	1.31–2.46	**< 0.001**	0.54	0.09–3.48	0.52
**Any sexually transmitted infections (STIs)**												
No	ref	ref		ref	ref		ref	ref		ref	ref	
Yes	1.94	1.40–2.69	**< 0.001**	0.52	0.21–1.33	0.18	1.66	1.21–2.26	**0.002**	0.24	0.03–1.63	0.14

NB: Each exposure was separately fitted in multivariable regression accounting for age, participant gender, and race/ethnicity. The SMM models adjusted for age and race/ethnicity.

The variable having anal sex was used in defining the SMM sample. Model could not be computed for diagnosis with gonorrhea and syphilis among SMM because nearly all the participant reported not missing PrEP.

The model for number of drinks containing alcohol per day in the last 3 months among SMM was unprecise (inflated estimates) due to missed PrEP distribution (0.00 %).

**Table 3:** Adjusted poisson regression with robust standard errors of the predictors of PrEP Use and missed PrEP due to alcohol among the Cisgender male and female (N = 592) and SMM and transgender individuals (N = 210).

Characteristics	Cisgender Male and Female						SMM and Transgender					
	Current PrEP Use			Missed PrEP due to Alcohol			Current PrEP Use			Missed PrEP doses due to alcohol		
			
	aRR	95 % CI	Sig.	aRR	95 % CI	Sig.	aRR	95 % CI	Sig.	aRR	95 % CI	Sig.
**Number of drinks containing alcohol per day in last 3 months**												
One (1) per day or less	ref	ref					ref	ref		ref	ref	
Two (2) – three (3) drinks per day	1.69	0.47–6.10	0.42				1.41	0.59–3.36	0.44	1.00	0.23–4.38	0.99
4 or more drinks per day	2.22	0.63–7.89	0.22				1.34	0.57–3.12	0.50	1.64	0.43–6.20	0.47
**Number of days drink alcohol last 3 months**												
Every day	ref	ref		ref	ref		ref	ref		ref	ref	
Less than 7 days/week	1.20	0.76–1.90	0.42	1.06	0.55–2.07	0.85	1.27	0.82–1.96	0.28	1.43	0.62–3.28	0.40
Less than 4 days per month	1.84	1.02–3.34	**0.04**	2.13	0.94–4.83	0.07	1.54	0.91–2.61	0.11	1.66	0.53–5.23	0.39
**Vaginal intercourse while under the influence of alcohol in the last 3 months**												
No	ref	ref		ref	ref		ref	ref		ref	ref	
Yes	0.25	0.18–0.36	**< 0.001**	0.68	0.37–1.24	0.21	0.59	0.43–0.82	**0.00**	1.70	0.95–3.04	0.08
**Condomless vaginal intercourse while under the influence of alcohol in the last 3 months**												
No	ref	ref		ref	ref		ref	ref		ref	ref	
Yes	0.57	0.43–0.77	**< 0.001**	1.51	0.90–2.51	0.12	0.77	0.55–1.07	0.11	2.02	1.14–3.59	**0.02**
**Anal intercourse while under the influence of alcohol in the last 3 months**												
No	ref	ref		ref	ref		ref	ref				
Yes	19.18	8.47–43.43	**< 0.001**	35.69	9.04–140.87	**< 0.001**	1.66	0.75–3.67	0.21			
**Condomless anal intercourse while under the influence of alcohol in the last 3 months**												
No	ref	ref		ref	ref		ref	ref		ref	ref	
Yes	11.46	6.29–20.86	**< 0.001**	21.55	7.91–58.68	**< 0.001**	1.72	1.04–2.85	**0.04**	2.18	0.73–6.46	0.16
**Cocaine use, number of days used in the last 30 days**	0.92	0.87–0.98	**0.01**	0.74	0.52–1.04	0.08	0.92	0.88–0.97	**0.00**	0.90	0.79–1.01	0.08
**Diagnosed with Gonorrhea**												
No	ref	ref					ref	ref		ref	ref	
Yes	1.99	1.29–3.08	**< 0.001**				1.54	1.12–2.12	**0.01**	0.27	0.05–1.44	0.13
**Diagnosed with Syphilis**												
No	ref	ref					ref	ref		ref	ref	
Yes	1.34	0.55–3.28	0.52				1.08	0.58–1.98	0.82	0.60	0.16–2.28	0.46
**Diagnosed with Chlamydia**												
No	ref	ref		ref	ref		ref	ref		ref	ref	
Yes	2.38	1.61–3.52	**< 0.001**	0.76	0.12–4.87	0.77	1.56	1.17–2.09	**0.00**	0.88	0.37–2.06	0.76
**Any sexually transmitted infections (STIs)**												
No	ref	ref		ref	ref		ref	ref		ref	ref	
Yes	2.18	1.54–3.09	**< 0.001**	0.31	0.05–2.14	0.24	1.53	1.16–2.03	**0.00**	0.44	0.17–1.16	0.09

Note: Each exposure was separately fitted in multiple regression accounting for age, gender and race/ethnicity.

## References

[R1] Agot K, Taylor D, Corneli AL, Wang M, Ambia J, Kashuba AD, Parker C, Lemons A, Malahleha M, Lombaard J, Van Damme L, 2015. Accuracy of self-report and pill-count measures of adherence in the FEM-PrEP clinical trial: implications for future HIV-prevention trials. AIDS Behav. 19 (5), 743–751. 10.1007/s10461-014-0859-z. 25100053 PMC4415940

[R2] Bandoli G, Palmsten K, Chambers CD, Jelliffe-Pawlowski LL, Baer RJ, Thompson CA, 2018. Revisiting the Table 2 fallacy: a motivating example examining preeclampsia and preterm birth. Paediatr. Perinat. Epidemiol 32 (4), 390–397. 10.1111/ppe.12474. 29782045 PMC6103824

[R3] Beesham I, Dovel K, Mashele N, Bekker L-G, Gorbach P, Coates TJ, Myer L, Joseph Davey DL, 2022. Correction to: barriers to oral HIV pre-exposure prophylaxis (PrEP) adherence among pregnant and post-partum women from Cape Town, South Africa. −3088 AIDS Behav. 26 (9), 3088. 10.1007/s10461-022-03676-8. 35316471 PMC8938734

[R4] Blumenthal J, Jain S, He F, Amico KR, Kofron R, Ellorin E, Stockman JK, Psaros C, Ntim GM, Chow K, Anderson PL, Haubrich R, Corado K, Moore DJ, Morris S, Landovitz RJ, 2021. Results from a pre-exposure prophylaxis demonstration project for at-risk Cisgender Women in the United States. Clin. Infect. Dis 73 (7), 1149–1156. 10.1093/cid/ciab328. 33864370 PMC8492205

[R5] Chan PA, Nunn A, van den Berg JJ, Cormier K, Sowemimo-Coker G, Napoleon SC, Arnold T, Moitra E, 2021. A randomized trial of a brief behavioral intervention for PrEP uptake among men who have sex with men at increased risk for HIV infection. J. Acquir Immune Defic. Syndr 87 (3), 937–943. 10.1097/qai.0000000000002671. 33734099 PMC8192434

[R6] Chou R, Evans C, Hoverman A, Sun C, Dana T, Bougatsos C, Grusing S, Korthuis PT, 2019. Preexposure prophylaxis for the prevention of HIV infection: evidence report and systematic review for the US preventive services task force. Jama 321 (22), 2214–2230. 10.1001/jama.2019.2591. 31184746

[R7] Dale SK, 2020. Using motivational interviewing to increase PrEP uptake among Black women at risk for HIV: an open pilot trial of MI-PrEP. J. Racial Ethn. Health Disparities 7 (5), 913–927. 10.1007/s40615-020-00715-9. 32078743

[R8] Fletcher OV, Beaver K, Austin EJ, van Draanen J, Edelman EJ, Williams EC, 2025. Perspectives and experiences regarding pre-exposure prophylaxis (PrEP) in a community sample of Veterans with unhealthy alcohol use: overall and across sexual orientation and gender identity. Addict. Sci. Clin. Pr 20 (1), 5. 10.1186/s13722-024-00533-y. PMC11773927 39876005

[R9] Friedman MR, Sang JM, Bukowski LA, Chandler CJ, Egan JE, Eaton LA, Matthews DD, Ho K, Raymond HF, Stall R, 2019. Prevalence and correlates of PrEP awareness and use among Black men who have sex with men and women (MSMW) in the United States. AIDS Behav. 23 (10), 2694–2705. 10.1007/s10461-019-02446-3. 30820849 PMC6713621

[R10] Gnardellis C, Notara V, Papadakaki M, Gialamas V, Chliaoutakis J, 2022. Overestimation of relative risk and prevalence ratio: misuse of logistic modeling. Diagnostics 12 (11). 10.3390/diagnostics12112851. PMC9689401 36428910

[R11] Goedel WC, Schneider JA, Hambrick HR, Kreski NT, Morganstein JG, Park SH, Mgbako O, Duncan DT, 2018. Are anal sex roles associated with preferences for pre-exposure prophylaxis administration modalities among men who have sex with men? Arch. Sex. Behav 47 (7), 2123–2133. 10.1007/s10508-017-1083-5. 29192368 PMC6008239

[R12] Groenwold RH, Klungel OH, Grobbee DE, Hoes AW, 2011. Selection of confounding variables should not be based on observed associations with exposure. Eur. J. Epidemiol 26 (8), 589–593. 10.1007/s10654-011-9606-1. 21796419 PMC3168755

[R13] Hardy C, Fairley CK, Ong JJ, Vodstrcil LA, Bradshaw CS, Snow A, Chow EPF, 2022. Drug and alcohol use with condomless anal sex among men who have sex with men in Melbourne, Australia: a retrospective data analysis from 2011 to 2017. Arch. Sex. Behav 51 (5), 2497–2507. 10.1007/s10508-021-01966-1. 34757603

[R14] Hojilla JC, Satre DD, Glidden DV, McMahan VM, Gandhi M, Defechereux P, Guanira JV, Mehrotra M, Grant RM, Carrico AW, 2019. Brief report: Cocaine use and pre-exposure prophylaxis: adherence, care engagement, and kidney function. J. Acquir Immune Defic. Syndr 81 (1), 78–82. 10.1097/qai.0000000000001972. 30730359 PMC6456371

[R15] Hutton HE, Lesko CR, Li X, Thompson CB, Lau B, Napravnik S, Mayer KH, Mathews WC, McCaul ME, Crane HM, Fredericksen RJ, Cropsey KL, Saag M, Christopoulos K, Chander G, 2019. Alcohol use patterns and subsequent sexual behaviors among women, men who have sex with men and men who have sex with women engaged in routine HIV care in the United States. AIDS Behav. 23 (6), 1634–1646. 10.1007/s10461-018-2337-5. 30443807 PMC6830881

[R16] Kalichman SC, Eaton L, 2017. Alcohol-antiretroviral interactive toxicity beliefs as a potential barrier to HIV pre-exposure prophylaxis among men who have sex with men. J. Int AIDS Soc 20 (1), 21534. 10.7448/ias.20.1.21534. 28715159 PMC5577742

[R17] Kalichman SC, Grebler T, Amaral CM, McNerney M, White D, Kalichman MO, Cherry C, Eaton L, 2014. Viral suppression and antiretroviral medication adherence among alcohol using HIV-positive adults. Int J. Behav. Med 21 (5), 811–820. 10.1007/s12529-013-9353-7. 24085706 PMC4283596

[R18] Kamitani E, Johnson WD, Wichser ME, Adegbite AH, Mullins MM, Sipe TA, 2020. Growth in proportion and disparities of HIV PrEP use among key populations identified in the United States national goals: systematic review and meta-analysis of published surveys. JAIDS J. Acquir. Immune Defic. Syndr 84 (4). 〈https://journals.lww.com/jaids/fulltext/2020/08010/growth_in_proportion_and_disparities_of_hiv_prep.7.aspx〉. 10.1097/QAI.0000000000002345 PMC8022293 32205721

[R19] Kamitani E, Wichser ME, Adegbite AH, Mullins MM, Johnson WD, Crouch P-C, Sipe TA, 2018. Increasing prevalence of self-reported HIV preexposure prophylaxis use in published surveys: a systematic review and meta-analysis. AIDS 32 (17), 2633–2635. 10.1097/qad.0000000000001983. 30096073 PMC6704375

[R20] Krakower D, Maloney KM, Powell VE, Levine K, Grasso C, Melbourne K, Marcus JL, Mayer KH, 2019. Patterns and clinical consequences of discontinuing HIV preexposure prophylaxis during primary care. J. Int AIDS Soc 22 (2), e25250. 10.1002/jia2.25250. 30768762 PMC6376610

[R21] Malotte CK, 2013. Brief risk-reduction counseling in clinical settings for HIV pre-exposure prophylaxis. Am. J. Prev. Med 44 (1), S112–S118. 10.1016/j.amepre.2012.09.033. 23253750

[R22] Marcus JL, Hurley LB, Hare CB, Nguyen DP, Phengrasamy T, Silverberg MJ, Stoltey JE, Volk JE, 2016. Preexposure prophylaxis for HIV prevention in a large integrated health care system: adherence, renal safety, and discontinuation. J. Acquir Immune Defic. Syndr 73 (5), 540–546. 10.1097/qai.0000000000001129. 27851714 PMC5424697

[R23] McCormack S, Dunn DT, Desai M, Dolling DI, Gafos M, Gilson R, Sullivan AK, Clarke A, Reeves I, Schembri G, Mackie N, Bowman C, Lacey CJ, Apea V, Brady M, Fox J, Taylor S, Antonucci S, Khoo SH, Gill ON, 2016. Pre-exposure prophylaxis to prevent the acquisition of HIV-1 infection (PROUD): effectiveness results from the pilot phase of a pragmatic open-label randomised trial. Lancet 387 (10013), 53–60. 10.1016/S0140-6736(15)00056-2. 26364263 PMC4700047

[R24] Oldfield BJ, Edelman EJ, 2021. Addressing unhealthy alcohol use and the hiv pre-exposure prophylaxis care continuum in primary care: a scoping review. AIDS Behav. 25 (6), 1777–1789. 10.1007/s10461-020-03107-6. 33219492 PMC8084877

[R25] Parsons JT, Rendina HJ, Lassiter JM, Whitfield TH, Starks TJ, Grov C, 2017. Uptake of HIV pre-exposure prophylaxis (PrEP) in a national cohort of gay and bisexual men in the United States. J. Acquir Immune Defic. Syndr 74 (3), 285–292. 10.1097/qai.0000000000001251. 28187084 PMC5315535

[R26] Qu D, Zhong X, Xiao G, Dai J, Liang H, Huang A, 2018. Adherence to pre-exposure prophylaxis among men who have sex with men: a prospective cohort study. Int J. Infect. Dis 75, 52–59. 10.1016/j.ijid.2018.08.006. 30125688

[R27] Sedgwick P., 2014. Relative risks versus odds ratios. BMJ Br. Med. J 348, g1407. 10.1136/bmj.g1407.

[R28] Sevelius JM, Poteat T, Luhur WE, Reisner SL, Meyer IH, 2020. HIV testing and PrEP use in a national probability sample of sexually active transgender people in the United States. J. Acquir Immune Defic. Syndr 84 (5), 437–442. 10.1097/qai.0000000000002403. 32692101 PMC7340231

[R29] Shuper PA, Joharchi N, Bogoch II, Loutfy M, Crouzat F, El-Helou P, Knox DC, Woodward K, Rehm J, 2020. Alcohol consumption, substance use, and depression in relation to HIV Pre-Exposure Prophylaxis (PrEP) nonadherence among gay, bisexual, and other men-who-have-sex-with-men. BMC Public Health 20 (1), 1782. 10.1186/s12889-020-09883-z. 33256651 PMC7706215

[R30] Shuper PA, Joharchi N, Varatharajan T, Bogoch II, Loutfy M, El-Helou P, Giolma K, Woodward K, Rehm J, 2024. Medical chart-reported alcohol consumption, substance use, and mental health issues in association with HIV pre-exposure prophylaxis (PrEP) nonadherence among gay, bisexual, and other men-who-have-sex-with-men. BMC Public Health 24 (1), 3487. 10.1186/s12889-024-20934-7. 39696067 PMC11658148

[R31] Shuper PA, Varatharajan T, Kinitz DJ, Gesink D, Joharchi N, Bogoch II, Loutfy M, Rehm J, 2022. Perceived influence of alcohol consumption, substance use, and mental health on PrEP adherence and condom use among PrEP-prescribed gay, bisexual, and other men-who-have-sex-with-men: a qualitative investigation. BMC Public Health 22 (1), 1875. 10.1186/s12889-022-14279-2. 36207757 PMC9540691

[R32] Siegler AJ, Mouhanna F, Giler RM, Weiss K, Pembleton E, Guest J, Jones J, Castel A, Yeung H, Kramer M, McCallister S, Sullivan PS, 2018. The prevalence of pre-exposure prophylaxis use and the pre-exposure prophylaxis-to-need ratio in the fourth quarter of 2017, United States. Ann. Epidemiol 28 (12), 841–849. 10.1016/j.annepidem.2018.06.005. 29983236 PMC6286209

[R33] StataCorp, 2023. Stata statistical software (Version Release 18.). StataCorp LLC.

[R34] Strong SH, Oldfield BJ, van den Berg JJ, Cole CA, Biegacki E, Ogbuagu O, Virata M, Chan PA, Edelman EJ, 2024. Perspectives on unhealthy alcohol use among men who have sex with men prescribed HIV pre-exposure prophylaxis: a qualitative study. Prev. Med Rep 37, 102553. 10.1016/j.pmedr.2023.102553. 38282665 PMC10810836

[R35] Sullivan PS, Sanchez TH, Zlotorzynska M, Chandler CJ, Sineath RC, Kahle E, Tregear S, 2020. National trends in HIV pre-exposure prophylaxis awareness, willingness and use among United States men who have sex with men recruited online, 2013 through 2017. J. Int AIDS Soc 23 (3), e25461. 10.1002/jia2.25461. 32153119 PMC7062633

[R36] Talbot D, Mésidor M, Chiu Y, Simard M, Sirois C, 2023. An alternative perspective on the robust poisson method for estimating risk or prevalence ratios. Epidemiology 34 (1), 1–7. 10.1097/ede.0000000000001544. 36125349

[R37] Teixeira da Silva D, Bouris A, Ramachandran A, Blocker O, Davis B, Harris J, Pyra M, Rusie LK, Brewer R, Pagkas-Bather J, Hotton A, Ridgway JP, McNulty M, Bhatia R, Schneider JA, 2021. Embedding a linkage to preexposure prophylaxis care intervention in social network strategy and partner notification services: Results from a pilot randomized controlled trial. J. Acquir Immune Defic. Syndr 86 (2), 191–199. 10.1097/qai.0000000000002548. 33109935 PMC8103968

[R38] Tran BX, Nguyen LT, Do CD, Nguyen QL, Maher RM, 2014. Associations between alcohol use disorders and adherence to antiretroviral treatment and quality of life amongst people living with HIV/AIDS. BMC Public Health 14, 27. 10.1186/1471-2458-14-27. 24411007 PMC3893525

[R39] Velloza J, Kemp CG, Aunon FM, Ramaiya MK, Creegan E, Simoni JM, 2020. Alcohol use and antiretroviral therapy non-adherence among adults living with HIV/AIDS in Sub-Saharan Africa: a systematic review and meta-analysis. AIDS Behav. 24 (6), 1727–1742. 10.1007/s10461-019-02716-0. 31673913 PMC7190427

[R40] Verinumbe T, Lucas GM, Zook K, Weir B, Landry M, Page KR, Sherman SG, Falade-Nwulia O, 2024. Associations of HIV pre-exposure prophylaxis (PrEP) indication, HIV risk perception and unwillingness to use PrEP among people who inject drugs in Baltimore, MD. Drug Alcohol Depend. Rep 13, 100288. 10.1016/j.dadr.2024.100288. 39498373 PMC11532811

[R41] Westreich D, Greenland S, 2013. The Table 2 fallacy: presenting and interpreting confounder and modifier coefficients. Am. J. Epidemiol 177 (4), 292–298. 10.1093/aje/kws412. 23371353 PMC3626058

[R42] Zou G., 2004. A modified poisson regression approach to prospective studies with binary data. Am. J. Epidemiol 159 (7), 702–706. 10.1093/aje/kwh090. 15033648

